# Journal of Veterinary Internal Medicine manuscript reviewers who critiqued in the 2022 calendar year

**DOI:** 10.1111/jvim.16632

**Published:** 2023-01-21

The Editorial Board thanks the following individuals for their time and effort spent in reviewing and critiquing manuscripts during the 2022 calendar year.

Aboellail, Tawfik; Colorado, USA

Acierno, Mark; Arizona, USA

Addie, Diane

Adin, Darcy; Florida, USA

Adkins, Pamela; Missouri, USA

Afonso, Tiago; Saskatchewan Canada

Aherne, Michael; Florida, USA

Aleman, Monica; California, USA

Allenspach, Karin; Iowa, USA

Al‐Nadaf, Sami; California, USA

Alves, Lisa; United Kingdom of Great Britain and Northern Ireland

Ames, Marisa; California, USA

Amorim, Irina; Portugal

Andrews, Frank; Louisiana, USA

Andruzzi, Melissa; California, USA

Angelos, John; California, USA

Applegate, Tanya; Colorado, USA

Archer, Joy; United Kingdom of Great Britain and Northern Ireland

Arenas‐Gamboa, Angela, USA

Armengou, Lara; Barcelona, Spain

Aroch, Itamar; Jerusalem, Israel

Atiee, Genna; Texas, USA

Atkins, Clarke; North Carolina, USA

Attipa, Charalampos; Merseyside, United Kingdom of Great Britain and Northern Ireland

Avery, Anne; Colorado, USA

Bach, Jan‐Peter; Germany

Bailey, Kirsten; Victoria, Australia

Balcomb, Christie; New Zealand

Balducci, Federica; Italy

Banse, Heidi; Louisiana, USA

Baral, Randolph; New South Wales Australia

Barker, Emily (Emi); USA

Barker, Lucy; United Kingdom of Great Britain and Northern Ireland

Barko, Patrick; Illinois, USA

Barnes Heller, Heidi; Wisconsin, USA

Baron Toaldo, Marco; Switzerland

Barr, Bonnie

Barrs, Vanessa; SAR China, Hong Kong

Bass, Luke; Colorado, USA

Batchelor, Daniel; United Kingdom of Great Britain and Northern Ireland

Bates, Nicola; United Kingdom of Great Britain and Northern Ireland

Bauquier, Jennifer; Victoria, Australia

Bayly, Warwick; Washington, USA

Beatty, Julia; Hong Kong

Beccati, Francesca; USA

Beckmann, Katrin; Zurich, Switzerland

Bedenice, Daniela; Massachusetts, USA

Beineke, Andreas; Germany

Bellone, Rebecca; California, USA

Beltran, Elsa; United Kingdom of Great Britain and Northern Ireland

Belz, Katie; Virginia, USA

Benchekroun, Ghita; Île‐de‐France, France

Bennett, Peter; New South Wales, Australia

Bentley, R. Timothy; Indiana, USA

Berghoff, Nora; Michigan, USA

Bernardini, Marco; Bologna, Italy

Berry, Kip; North Carolina, USA

Bertin, Francois‐Rene; Queensland, Australia

Besche, Beatrice; Aquitaine, France

Betting, Adeline; Switzerland

Bianco, Domenico; California, USA

Bierlein, Metzere; California, USA

Billen, Frédéric; Belgium

Birettoni, Francesco; Italy

Bissett, Sally; North Carolina, USA

Blois, Shauna; Ontario, Canada

Boileau, Melanie; Oklahoma, USA

Boland, Lara; New South Wales, Australia

Bonelli, Francesca; Italy

Bonelli, Marilia; Ohio, USA

Bordin, Angela; Texas, USA

Boretti, Felicitas; Switzerland

Borges, Alexandre; Sao Paulo, Brazil

Borio, Stefano

Bosch, Luis; Bellaterra, Spain

Boswood, Adrian; Hertfordshire, United Kingdom of Great Britain and Northern Ireland

Boudreaux, Bonnie; Louisiana, USA

Bove, Christina; Colorado, USA

Boyd, Corrin; Western Australia, Australia

Boyé, Pierre; France

Boyle, Ashley; Pennsylvania, USA

Bracker, Kiko; Massachusetts, USA

Brainard, Benjamin; Georgia, USA

Breheny, Craig; United Kingdom of Great Britain and Northern Ireland

Brewer, Lawrence; USA

Brockman, Daniel James; USA

Brooks, Marjory; New York, USA

Bryan, Jeffrey; Missouri, USA

Buczinski, Sebastien; Quebec, Canada

Bullone, Michela; Quebec, Canada

Burge, Rhonda; Ohio, USA

Burgess, Brandy; Georgia, USA

Burns, Teresa; Ohio, USA

Burton, Jenna; Colorado, USA

Byrne, Barbara; California, USA

Byron, Julie; Ohio, USA

Caldwell, Marc; Tennessee, USA

Canal, Sara; Ancona, Italy

Canapp, Sherman; USA

Cardoso, Luis; Portugal

Carey, Stephan; Michigan, USA

Case, J. Brad; Florida, USA

Castagnetti, Carolina; Italy

Catalfamo, Jim; New York, USA

Catchpole, Brian; United Kingdom of Great Britain and Northern Ireland

Cavalleri, Jessika; Austria

Center, Sharon; New York, USA

Ceplecha, Vaclav; Czech Republic

Chacar, Fernanda; Sao Paulo, Brazil

Chako, Clemence; Arizona, USA

Chalhoub, Serge; Alberta, Canada

Chamorro, Manuel; Alabama, USA

Chandler, Marjorie (Marge); United Kingdom of Great Britain and Northern Ireland

Charalambous, Marios; Germany

Chew, Dennis; Ohio, USA

Chidlow, Hayley; Georgia, USA

Childress, Michael; Indiana, USA

Chiocchetti, Roberto; Italy

Choi, Eunju; California, USA

Chun, Ruthanne; Wisconsin, USA

Clercx, Cecile; Belgium

Clow, Katie; Ontario, Canada

Collinet, Audrey; Florida, USA

Comazzi, Stefano; Italy

Cordella, Alessia; Emilia‐Romagna, Italy

Corradini, Ignacio; Valencia, Spain

Cosford, Kevin; Saskatchewan, Canada

Costa, Lais; California, USA

Costa, Marcio; Quebec, Canada

Cote, Etienne; Prince Edward Island, Canada

Couetil, Laurent; Indiana, USA

Couto, Guillermo; Ohio, USA

Cowgill, Larry; California, USA

Cozzi, Francesca; Italy

Credille, Brent; Georgia, USA

Cridge, Harry; Michigan, USA

Cullen, John; North Carolina, USA

Cunningham, Suzanne; Massachusetts, USA

Cuq, Benoît; Ireland

Curran, Kaitlin; Oregon, USA

Czerwik, Adriana; Germany

Daminet, Sylvie; Belgium

Dandrieux, Julien; Victoria, Australia

Davidow, Beth; Washington, USA

Davis, Jennifer; Virginia, USA

de Laat, Melody; Queensland, Australia

De Laforcade, Armelle; Massachusetts, USA

Dear, Jonathan; California, USA

Decaro, Nicola; Bari, Italy

Decloedt, Annelies; Belgium

DeDonder, Keith; Maryland, USA

Defarges, Alice; Ontario, Canada

DeFrancesco, Teresa; North Carolina, USA

Dehghanpir, Shannon; Louisiana, USA

Del Baldo, Francesca; Italy

DeMonaco, Stefanie; Virginia, USA

Depenbrock, Sarah; California, USA

Deshuillers, Pierre; France

Deveau, Michael; Texas, USA

Dittmer, Keren; New Zealand

Divers, Thomas; New York, USA

Dodds, W. Jean; California, USA

Dreier, Jens; Germany

Driver, Colin; USA

Dunn, Marilyn; Quebec, Canada

Durham, Andy; Hampshire, United Kingdom of Great Britain and Northern Ireland

Duval, Dawn

Eades, Susan; Texas, USA

Eberhardt, Christina; South Africa

El‐Hage, Charles; Victoria, Australia

Elliott, James; United Kingdom of Great Britain and Northern Ireland

Elliott, Jonathan; United Kingdom of Great Britain and Northern Ireland

Elsohaby, Ibrahim; Prince Edward Island, Canada

Epstein, Steven; California, USA

Erdahl, Erol

Erol, Erdal; Kentucky, USA

Estell, Krista; Virginia, USA

Evans, Samantha; Ohio, USA

Fadda, Angela Avon; United Kingdom of Great Britain and Northern Ireland

Faller, Kiterie; United Kingdom of Great Britain and Northern Ireland

Farke, Daniela; Germany

Farré Mariné, Alba; Valencia, Spain

Farrell, Kate; California, USA

Fauber, Amy; Indiana, USA

Feary, Darien; New South Wales Australia

Fellman, Claire; Massachusetts, USA

Ferri, Filippo; Novara, Italy

Fielding, Langdon; California, USA

Finch, Natalie; United Kingdom of Great Britain and Northern Ireland

Fink‐Gremmels; Johanna, Netherlands

Finnen, Andrea; Ontario, Canada

Finno, Carrie; California, USA

Fischer, Andrea; Bayern, Germany

Fischetti, Anthony; USA

Fjeldborg, Julie; Copenhagen, Denmark

Flanagan, John; Occitanie, France

Flegel, Thomas; Germany

Flesner, Brian; Pennsylvania, USA

Floyd, Emily

Foss, Kari; Illinois, USA

Foster, Derek; North Carolina, USA

Foster, Jonathan (JD); District of Columbia, USA

Fouché, Nathalie; Switzerland

Fousse, Samantha; California, USA

Foy, Daniel; Arizona, USA

Franca, Monica; USA

Francey, Thierry; Switzerland

Francoz, David; Quebec, Canada

Freeman, Lisa; Massachusetts, USA

Freeman, Paul; United Kingdom of Great Britain and Northern Ireland

Fries, Ryan; Illinois, USA

Fritz, Heather; California, USA

Fulkerson, Christopher; Indiana, USA

Furr, Martin; Oklahoma, USA

Furrow, Eva; Minnesota, USA

Gagnon, Allison; California, USA

Galban, Evelyn; Pennsylvania, USA

Gallagher, Alexander; Florida, USA

Gamsjaeger, Lisa; USA

Garabed, Rebecca; Ohio, USA

Garcia Sancho, Mercedes; Madrid, Spain

Garcia, Gabriel; Florida, USA

Garcia, Ryan; California, USA

Garner, Bridget

Gaschen, Frederic; Louisiana, USA

Gay, John; Washington, USA

Geddes, Rebecca; United Kingdom of Great Britain and Northern Ireland

Gentile, Arcangelo; Italy

Gentile, Jessica; Maine, USA

German, Alexander; Cheshire, United Kingdom of Great Britain and Northern Ireland

Giebels, Felix; Switzerland

Gilbertie, Jessica; Virginia, USA

Gilliam, John; Oklahoma, USA

Gilor, Chen; Florida, USA

Gilsenan, William; Kentucky, USA

Giuffrida, Michelle; California, USA

Glanemann, Barbara; United Kingdom of Great Britain and Northern Ireland

Gnirs, Kirsten; France

Gold, Jenifer; Washington, USA

Gomes, Sergio Andrade; Derbyshire, United Kingdom of Great Britain and Northern Ireland

Gomez, Diego; Ontario, Canada

Goncalves, Rita; United Kingdom of Great Britain and Northern Ireland

Gookin, Jody; North Carolina, USA

Gori, Eleonora; Italy

Gostelow, Ruth; Hertfordshire, United Kingdom of Great Britain and Northern Ireland

Gotthelf, Louis; Alabama, USA

Granger, Nicolas; Bristol, United Kingdom of Great Britain and Northern Ireland

Grant, David; Virginia, USA

Greensmith, Tom

Griffin IV, John

Grobman, Megan; Alabama, USA

Grouzmann, Eric; Switzerland

Guedes, Alonso; California, USA

Guess, Sarah; Washington, USA

Guglielmini, Carlo; Italy

Gull, Tamara; Missouri, USA

Gustafson, Daniel; Colorado, USA

Gutierrez‐Quintana, Rodrigo; United Kingdom of Great Britain and Northern Ireland

Hahn, Caroline; Scotland, United Kingdom of Great Britain and Northern Ireland

Haines, Jillian; Washington, USA

Hall, Alison; United Kingdom of Great Britain and Northern Ireland

Hall, Jean; Oregon, USA

Hansen, Katherine; California, USA

Hanzlicek, Andrew; Indiana, USA

Harcourt‐Brown, Tom; Avon, United Kingdom of Great Britain and Northern Ireland

Hardefeldt, Laura; Victoria, Australia

Harkin, Kenneth; Kansas, USA

Harris, Autumn; Florida, USA

Hart, Kelsey; Georgia, USA

Harvey, John; Florida, USA

Hasegawa, Daisuke; Tokyo, Japan

Havel, Peter; California, USA

Hawkins, Eleanor; North Carolina, USA

Hedgespeth, Barry; North Carolina, USA

Heilmann, Romy; Saxony, Germany

Hepburn, Richard; Gloucestershire, United Kingdom of Great Britain and Northern Ireland

Herbert, Eleanor; United Kingdom of Great Britain and Northern Ireland

Herndon, Aaron; Queensland Australia

Herrtage, Michael; United Kingdom of Great Britain and Northern Ireland

Heseltine, Johanna; Texas, USA

Hess, Rebecka; Pennsylvania, USA

Hesselkilde, Eva; Denmark

Hewetson, Michael; Hertfordshire, United Kingdom of Great Britain and Northern Ireland

Hezzell, Melanie; United Kingdom of Great Britain and Northern Ireland

Hill, Tracy; Minnesota, USA

Hocker, Samuel; Kansas, USA

Hon, Stephanie; New York, USA

Hopster‐Iversen, Charlotte; Denmark

Howerth, Elisabeth; Georgia, USA

Hsue, Weihow; New York, USA

Huber, Laura; Alabama, USA

Hughes, Kris; New South Wales, Australia

Humm, Karen; United Kingdom of Great Britain and Northern Ireland

Husnik, Roman; Ontario, Canada

Hutchinson, Holden; Colorado, USA

Irwin, Peter, Western Australia, Australia

Ito, Daisuke; Kanagawa, Japan

Ivester, Kathleen; Indiana, USA

Jablonski, Sara; Michigan, USA

Jacob, Megan E.; USA

James, Fiona; Ontario, Canada

Jeffery, Nicholas; Texas, USA

Jepson, Rosanne; Herts, United Kingdom of Great Britain and Northern Ireland

Johannes, Chad; Iowa, USA

Johns, Imogen; Herts, United Kingdom of Great Britain and Northern Ireland

Johnson, Amy, Pennsylvania, USA

Johnson, Amy; Leigh, USA

Johnson, Lynelle; California, USA

Johnston, Andrea; Louisiana, USA

Jokinen‐Pääkkönen, Tarja; Finland

Jose‐Cunilleras, Eduard; Barcelona, Spain

Kaplan, Joanna; California, USA

Kather, Stefanie; Germany

Katherman, Anne; Virginia, USA

Kathrani, Aarti; United Kingdom of Great Britain and Northern Ireland

Katz, Martin; Missouri, USA

Keen, John; Scotland, United Kingdom of Great Britain and Northern Ireland

Keene, Bruce; North Carolina, USA

Kellihan, Heidi; Wisconsin, USA

Kendall, Allison; North Carolina, USA

Kent, Michael; California, USA

Kerl, Marie; Missouri, USA

Khan, Zohra; United Kingdom of Great Britain and Northern Ireland

Kidd, Linda; California, USA

Kiene, Frederik; Germany

Kittleson, Mark; California, USA

Kleber, Kara

Knauer, Whitney; USA

Knowles, Edward; USA

Knych, Heather; California, USA

Koch, Christoph, Berne, Switzerland

Komaromy, Andras; Michigan, USA

Kooistra, Hans; The Netherlands

Kook, Peter; Zurich, Switzerland

Kopecny, Lucy; California, USA

Kopper, Jamie; Washington, USA

Korchia, Jeremie; Colorado, USA

Kornya, Matthew, Ontario, Canada

Kortz, Gregg; USA

Krogh, Anne; USA

KuKanich, Kate; Kansas, USA

Kurosawa, Tsumugi (Anne); United Kingdom of Great Britain and Northern Ireland

Labato, Mary Anna; Massachusetts, USA

Lacorcia, Lauren; Victoria, Australia

Ladlow, Jane; United Kingdom of Great Britain and Northern Ireland

Laflamme, Dorothy (Dottie); Virginia, USA

Langlois, Daniel; Michigan, USA

Lapsley, Janis; Ohio, USA

Larsen, Jennifer; California, USA

Larson, Martha; USA

Lascelles, Duncan; North Carolina, USA

Lashnits, Erin; North Carolina, USA

Lathan, Patty; Mississippi, USA

Laver, Travis; Georgia, USA

Lawhon, Sara; Texas, USA

Lazzerini, Kali; Glasgow, United Kingdom of Great Britain and Northern Ireland

Leal, Rodolfo; Portugal

LeBlanc, Amy; Maryland, USA

Leclere, Mathilde; Quebec Canada

Lecollinet, Sylvie; France

Lee, Ya‐Jane; Taiwan

Leeb, Tosso; Switzerland

Leeper, Haley; Oregon, USA

Leguillette, Renaud; Alberta, Canada

Leisewitz, Andrew; Alabama, USA

Levine, Jonathan; Texas, USA

Li, Ronald; California, USA

Lidbury, Jonathan; Texas, USA

Lilliehöök, Inger; Sweden

Ljungvall, Ingrid; Sweden

Lobetti, Remo; South Africa

Londono, Leo; Florida, USA

Long, Sam; Victoria, Australia

Lopez, Brina

Lorenz, Ingrid; Germany

Lourenço, Bianca; Georgia, USA

Loy, Dustin; Nebraska, USA

Luckschander‐Zeller, Nicole; Kragujevac, Austria

Luethy, Daniela; Pennsylvania, USA

Lulich, Jody; Minnesota, USA

Lund, Heidi; USA

Lunn, Katharine; North Carolina, USA

Lurie, David; New South Wales Australia

Lynch, Alex; North Carolina, USA

Lyon, Shane; Kansas, USA

Lyons, Leslie; USA

MacKillop, Edward; Pennsylvania, USA

Mackin, Andrew; Mississippi, USA

MacNeill, Amy; Colorado, USA

Mahony, Orla; Massachusetts, USA

Mai, Wilfried; Pennsylvania, USA

Mair, Tim; Kent, United Kingdom of Great Britain and Northern Ireland

Malik, Richard; New South Wales, Australia

Mallicote, Martha; Florida, USA

Manchester, Alison; Colorado, USA

Manguin, Estelle; Quebec, Canada

Mansfield, Caroline; Victoria, Australia

Manzi, Timothy; Pennsylvania, USA

Marconato, Laura; Emilia‐Romagna, Italy

Marioni‐Henry, Katia; Midlothian, United Kingdom of Great Britain and Northern Ireland

Markovic, Lauren; Georgia, USA

Marks, Stanley; California, USA

Marolf, Angela; Colorado, USA

Marsilio, Sina; California, USA

Martinez, Jennifer; Florida, USA

Massie, Shannon; Alberta, Canada

Mathews, Karol; Ontario, Canada

Mauler, Daniela; Missouri, USA

Mayhew, Joe; New Zealand

Mazan, Melissa; Massachusetts, USA

McDonnell, John; Maryland, USA

McFarlane, Dianne; Oklahoma, USA

McGowan, Catherine (Cathy); United Kingdom of Great Britain and Northern Ireland

McGrath, Stephanie; USA

Mchale, Brittany; Georgia, USA

McMichael, Maureen; Alabama, USA

McNabb, Bret; California, USA

Meier, Valeria; Zurich, Switzerland

Mellersh, Cathryn; Suffolk, United Kingdom of Great Britain and Northern Ireland

Meneses, Felix; Switzerland

Menzies‐Gow, Nicola; Herts, United Kingdom of Great Britain and Northern Ireland

Messam, Locksley

Metzger, Julia; Germany

Meuten, Donald; North Carolina, USA

Meyerhoff, Nina; Lower Saxony, Germany

Mickelson, James

Mickelson, Megan; Missouri, USA

Milne, Marjorie; Victoria, Australia

Mintz, Shana; New York, USA

Miranda, M.; Lugo, Spain

Mooney, Carmel; Ireland

Moore, George; Indiana, USA

Moore, Sarah; Ohio, USA

Moore‐Colyer, Meriel; United Kingdom of Great Britain and Northern Ireland

Morita, Tomoya; Japan

Morresey, Peter; Kentucky, USA

Motta, Jean‐Paul; France

Mukkänen, Anna; Finland

Muñana, Karen; North Carolina, USA

Murakami, Keiko; Iowa, USA

Murtagh, Kevin; Ireland

Murthy, Vishal; Washington, USA

Nabity, Mary; Texas, USA

Nafe, Laura; Missouri, USA

Nagy, Dusty; Texas, USA

Nakamura, Reid; California, USA

Nath, Laura; South Australia Australia

Navarro, Mauricio; USA

Naylor, Rosie; Hertfordshire, United Kingdom of Great Britain and Northern Ireland

Nessler, Jasmin; Germany

Newman, Ashleigh; New York, USA

Nielsen, Lise; Denmark

Niessen, Stijn; Herts, United Kingdom of Great Britain and Northern Ireland

Nishida, Hidetaka; Japan

Nocera, Irene; Livorno, Italy

Nolan, Michael; USA

O'Brien, Dennis; Missouri, USA

Ohmori, Keitaro; Tokyo, Japan

Ojeda, Javier; Chile

Olby, Natasha; North Carolina, USA

Ollivett, Terri; Wisconsin, USA

Orvalho, Joao; California, USA

O'Sullivan, Lynne; Prince Edward Island, Canada

Otte, Corma; Utrecht, The Netherlands

Outerbridge, Catherine; California, USA

Overall, Karen

Packer, Rowena; United Kingdom of Great Britain and Northern Ireland

Paepe, Dominique; Belgium

Pakozdy, Akos; Austria

Papich, Mark; North Carolina, USA

Paraschou, Georgios; Saint Kitts and Nevis

Pardon, Bart; Belgium

Pariaut, Romain; New York, USA

Park, Hee‐Myung

Parker, Rell; Virginia, USA

Parker, Valerie; Ohio, USA

Parkinson, Nicholas; Midlothian, United Kingdom of Great Britain and Northern Ireland

Passler, Thomas; Alabama, USA

Paulin, Mathieu; Saskatchewan, Canada

Pavelski, Mariana; Brazil

Pawlak, Aleksandra; Dolnośląskie, Poland

Pazzi, Paolo; Gauteng, South Africa

Peaston, Anne; South Australia, Australia

Penning, Louis; Utrecht, The Netherlands

Percival, Aaron; New York, USA

Perego, Manuela; Varese, Italy

Pesavento, Patricia; California, USA

Peters, Laureen Michèle; Switzerland

Petersen, Jessica; Nebraska, USA

Pezzanite, Lynn; Colorado, USA

Piazza, S.

Pihl, Tina; Denmark

Pilla, Rachel; Texas, USA

Pithua, Patrick; Virginia, USA

Pollard, Rachel; California, USA

Pomrantz, Jill; Maine, USA

Poulsen, Keith; Wisconsin, USA

Pratt‐Phillips, Shannon; USA

Prieto, Jennifer; New York, USA

Pringle, John; Sweden

Pritchard, Jessica; Wisconsin, USA

Provencher, Michele; Ohio, USA

Pruemmer, Julia; Switzerland

Prutton, James; Hampshire, United Kingdom of Great Britain and Northern Ireland

Quimby, Jessica; Ohio, USA

Rademacher, Nathalie; Louisiana, USA

Raidal, Sharanne; New South Wales Australia

Raj, Jen; Essex, United Kingdom of Great Britain and Northern Ireland

Rajamäki, Minna; Finland

Ramsauer, Anna Sophie; Austria

Reagan, Krystle; California, USA

Reed, Stephen; Kentucky, USA

Reetz, Jennifer; Pennsylvania, USA

Refetoff, Samuel; USA

Refsal, Kent; Michigan, USA

Reinhart, Jennifer; Illinois, USA

Renaud, David; Ontario Canada

Rendle, David; Hampshire, United Kingdom of Great Britain and Northern Ireland

Reppert, Emily; Kansas, USA

Richard, Eric; France

Richter, Angelika; Germany

Ridyard, Alison; United Kingdom of Great Britain and Northern Ireland

Riedesel, Elizabeth; Iowa, USA

Riese, Franziska; Germany

Rigas, Konstantinos; United Kingdom of Great Britain and Northern Ireland

Riihimaki, Miia; Sweden

Rings, Lindsey; Ohio, USA

Roche‐Catholy, Marine; Belgium

Rogatko, Cleo; New York, USA

Rohdin, Cecilia; Sweden

Romano, Megan; Kentucky, USA

Rose, Jeremy; United Kingdom of Great Britain and Northern Ireland

Ross, Linda; Massachusetts, USA

Ross, Sheri; Canada

Rossmeisl, John; Virginia, USA

Roy, Marie‐France; Alberta, Canada

Royaux, E.; USA

Rozanski, Elizabeth; Massachusetts, USA

Ruaux, Craig; New South Wales, Australia

Ruby, Rebecca; Kentucky, USA

Rush, John; Massachusetts, USA

Rütgen, Barbara; Kragujevac, Austria

Ryan, Clare; Georgia, USA

Rylander, Helena Wisconsin, USA

Saker, Korinn; North Carolina, USA

Santangelo, Kelly; USA

Santifort, Koen; The Netherlands

Saunders, Ashley; Texas, USA

Savevski, Victor; Milano, Italy

Schaaf, Cecilia; North Carolina, USA

Schaer, Michael; Florida, USA

Scharf, Valery; North Carolina, USA

Schatzberg, Scott; Texas, USA

Schenck, Patricia Michigan, USA

Schermerhorn, Thomas; USA

Schnabel, Lauren; North Carolina, USA

Schoeman, Johan; Gauteng, South Africa

Schoster, Angelika; Switzerland

Schott II, Harold; Michigan, USA

Schulz, Bianka; Germany

Schweighauser, Ariane; Bern, Switzerland

Schweizer, Daniela; Bern, Switzerland

Scott, Katherine; USA

Scott, Mike

Scott‐Moncrieff, Catharine; Indiana, USA

Segev, Gilad; Israel

Sellon, Rance; Washington, USA

Selmic, Laura; Ohio, USA

Seo, Joonbum (Joon); New Zealand

Serrano, Goncalo; Belgium

Sewell, Adrian; Germany

Shamir, Merav; Israel

Shamoun, John; North Carolina, USA

Sharp, Claire; Western Australia, Australia

Shelton, G. Diane; California, USA

Shiel, Robert; Australia

Shihab, Nadia

Shores, Andy; Mississippi, USA

Shropshire, Sarah; Colorado, USA

Sierra‐Rodriguez, Tamara; Alabama, USA

Sillence, Martin; Queensland, Australia

Silverstein, Deborah; Pennsylvania, USA

Silvestri, Serenella; Italy

Siso, Silvia; California, USA

Slack, Joann; USA

Slead, Tanner; North Carolina, USA

Sleeper, Margaret (Meg); Florida, USA

Slovak, Jennifer; Wyoming, USA

Smith, Geof; North Carolina, USA

Smith, Jo; Georgia, USA

Smith, Stephanie; Michigan, USA

Snyder, Jessica; Washington, USA

Soebbeler, Franz; Niedersachsen Germany

Sojka Kritchevsky, Janice; Indiana, USA

Soler Arias, Elber; Argentina

Soma, Lawrence; Pennsylvania, USA

Spada, Eva; Italy

Spadavecchia, Claudia; Switzerland

Specht, Andrew; Florida, USA

Spillmann, Thomas; Finland

Spriet, Mathieu California, USA

Stabile, Fabio; United Kingdom of Great Britain and Northern Ireland

Stammeleer, Lisa; Belgium

Stander, Nerissa; Western Australia, Australia

Stee, Kimberley; United Kingdom of Great Britain and Northern Ireland

Steffen, Frank; Switzerland

Stepien, Rebecca; Wisconsin, USA

Stern, Joshua; California, USA

Steuer, Ashley; Texas, USA

Stieger‐Vanegas, Susanne; Oregon, USA

Stockman, Jonathan; New York, USA

Strain, George; Louisiana, USA

Suárez‐Esquivel, Marcela; Costa Rica

Suchodolski, Jan; Texas, USA

Sullins, Ken; Arizona, USA

Sullivan, Stacey; Australia

Summers, Stacie; Oregon, USA

Sutherland, Katja; Ontario, Canada

Sutton, David; Scotland, United Kingdom of Great Britain and Northern Ireland

Sykes, B. W.; USA

Sykes, Jane; California, USA

Syme, Harriet; Herts, United Kingdom of Great Britain and Northern Ireland

Taeymans, Olivier; Massachusetts, USA

Takai, Shinji; Towada, Japan

Tamborini, Alice; Ireland

Tauro, Anna; United Kingdom of Great Britain and Northern Ireland

Taylor, Collette; United Kingdom of Great Britain and Northern Ireland

Taylor, Sandra; Indiana, USA

Taylor‐Brown, Frances; United Kingdom of Great Britain and Northern Ireland

Teske, Erik; The Netherlands

Theron, Marie‐Laure; France

Thomas, William; Tennessee, USA

Thomason, John; Mississippi, USA

Thomovsky, Stephanie; Indiana, USA

Thompson, Craig; Indiana, USA

Thompson, Dan; United Kingdom of Great Britain and Northern Ireland

Thompson, Margret; New York, USA

Thomson, Christine; Australia

Tidholm, Anna; Sweden

Tivers, Michael; United Kingdom of Great Britain and Northern Ireland

Tjostheim, Sonja; Wisconsin, USA

Toedebusch, Christine; California, USA

Tolbert, M. Katherine; Texas, USA

Toombs‐Ruane, Leah; Manawatu‐Wanganui New Zealand

Toresson, Linda; Finland

Toribio, Ramiro; Ohio, USA

Trepanier, Lauren; Wisconsin, USA

Twedt, David; Colorado, USA

Ueda, Yu; North Carolina, USA

Urbano, Tara

Vail, David; Wisconsin, USA

van den Boom, Robin; The Netherlands

van Eps, Andrew; Pennsylvania, USA

van Loon, Gunther; Belgium

van Steenbeek, Frank; The Netherlands

Van Wettere, Arnaud; Utah, USA

Vandevelde, Marc; Switzerland

Vanhaesebrouck, An; Cambridgeshire, United Kingdom of Great Britain and Northern Ireland

VanHoy, Grace; California, USA

VanWormer, Elizabeth; Nebraska, USA

Vernau, William; California, USA

Viall, Austin; California, USA

Viitanen, Sanna; Finland

Vinardell, Tatiana; Qatar

Vishkautsan, Polina; Arizona, USA

Visser, Lance; Colorado, USA

Vite, Charles; Pennsylvania, USA

Viviano, Katrina; Wisconsin, USA

Voges, Andra; Texas, USA

Wachowiak, Ian; Colorado, USA

Waldridge, Bryan; Mississippi, USA

Walker, Julie; Wisconsin, USA

Wallace, Mandy

Walsh, Koranda; Pennsylvania, USA

Wang‐Leandro, Adriano; Germany

Ward, Jessica; Iowa, USA

Wardrop, Jane; Washington, USA

Watson, Penny; United Kingdom of Great Britain and Northern Ireland

Webb, Craig; Colorado, USA

Webster, Cynthia; Massachusetts, USA

Weese, J (Scott); Ontario, Canada

Wehner, Astrid; Germany

Weishaar, Kristen; Colorado, USA

Weisse, Chick; New York, USA

Werners, Arno; St. George, Grenada

Wessmann, Annette; United Kingdom of Great Britain and Northern Ireland

Whitcomb, Mary Beth; California, USA

Whitehouse, William; Kansas, USA

Whitfield‐Cargile, Canaan; Georgia, USA

Wiggen, Kelly; Missouri, USA

Wilkins, Pamela; Illinois, USA

Williams, Deniece; California, USA

Williams, Diane; California, USA

Williams, Timothy (Tim); United Kingdom of Great Britain and Northern Ireland

Wilshaw, Jenny; Hertfordshire, United Kingdom of Great Britain and Northern Ireland

Winger, Kathryn; Michigan, USA

Winston, Jenessa; Ohio, USA

Wise, Jessica; New South Wales, Australia

Wismer, Tina; New York, USA

Woldemeskel, Moges; Georgia, USA

Wolf, Jacob; Florida, USA

Wolff, Ewan; Florida, USA

Wood, James; California, USA

Wood, Michael; Wisconsin, USA

Woods, Glynn; United Kingdom of Great Britain and Northern Ireland

Woods, Katharine; Saskatchewan, Canada

Worthing, Kate; Arizona, USA

Wotman, Kathryn; Colorado, USA

Wright, Kathy; Ohio, USA

Xenoulis, Panagiotis; Texas, USA

Yang, Vicky; Massachusetts, USA

York, Daniel; California, USA

